# 

**Published:** 1904-10-15

**Authors:** 


### ANNOUNCEMENTS.

THE Ohio State Board of Dental Examiners will hold its regular semi-annual meeting at Columbus, Ohio, in the Hartman Hotel, on November 29th, and 30th, and December 1st. Applications for examination should be filed with the secretary before November 20th.

For full information, address,

DR. H. C. BROWN, *Sec'y*,
185 East State St., Columbus, O.

THE S. S. White Dental Manufacturing Co. issued a miniature booklet as a souvenir of the International Congress, containing a history of the *Dental Cosmos* and the development of the great success of this company. It is a handsomely printed little pamphlet and contains good portraits of all who have contributed largely to the success of the house.

THE Ohio State Dental Society meets the first week in December. It is the desire of the chairman of the Clinic Committee to present a strong list of clinics, and would therefore request that any one desiring to present a clinic for this meeting should make it known at the earliest practicable moment. Address Dr. C. A. Hawley, 185 East State St., Columbus, O.

WISCONSIN State Dental Society elected as its officers for the next year: President, H. T. Sackett; Vice Presidents, E. C. Oviatt, and Percy B. Wright; Secretary, W. H. Mueller, Madison, Treasurer, A. Gropper, The next meeting will be held at Oshkosh in July.

THERE will be no meeting of the South-western Michigan Dental Society this fall, because of the many other important meetings conflicting.

